# Developmental patterns in human blood–brain barrier and blood–cerebrospinal fluid barrier ABC drug transporter expression

**DOI:** 10.1007/s00418-020-01884-8

**Published:** 2020-05-24

**Authors:** L. F. M. Verscheijden, A. C. van Hattem, J. C. L. M. Pertijs, C. A. de Jongh, R. M. Verdijk, B. Smeets, J. B. Koenderink, F. G. M. Russel, S. N. de Wildt

**Affiliations:** 1 Department of Pharmacology and Toxicology, Radboud University Medical Center, Institutes for Molecular Life and Health Sciences, Nijmegen, The Netherlands; 2 Section Neuropathology and Ophthalmic Pathology, Department of Pathology, Erasmus MC University Medical Center, Rotterdam, The Netherlands; 3 Department of Pathology, Radboud University Medical Center, Nijmegen, The Netherlands; 4 Intensive Care and Department of Paediatric Surgery, Erasmus MC-Sophia Children’s Hospital, Rotterdam, The Netherlands

**Keywords:** Blood–brain barrier, Blood–cerebrospinal fluid barrier, ATP-binding cassette transporters, Brain pharmacokinetics, Fetus, Child

## Abstract

When drugs exert their effects in the brain, linear extrapolation of doses from adults could be harmful for children as the blood–brain barrier (BBB) and blood–CSF barrier (BCSFB) function is still immature. More specifically, age-related variation in membrane transporters may impact brain disposition. As human data on brain transporter expression is scarce, age dependent [gestational age (GA), postnatal age (PNA), and postmenstrual age (PMA)] variation in immunohistochemical localization and staining intensity of the ABC transporters P-glycoprotein (Pgp), breast cancer resistance protein (BCRP), and multidrug resistance-associated proteins 1, 2, 4, and 5 (MRP1/2/4/5) was investigated. Post mortem brain cortical and ventricular tissue was derived from 23 fetuses (GA range 12.9–39 weeks), 17 neonates (GA range 24.6–41.3 weeks, PNA range 0.004–3.5 weeks), 8 children (PNA range 0.1–3 years), and 4 adults who died from a wide variety of underlying conditions. In brain cortical BBB, immunostaining increased with age for Pgp and BCRP, while in contrast, MRP1 and MRP2 staining intensity appeared higher in fetuses, neonates, and children, as compared to adults. BCSFB was positively stained for Pgp, MRP1, and MRP2 and appeared stable across age, while BCRP was not detected. MRP4 and MRP5 were not detected in BBB or BCSFB. In conclusion, human BBB and BCSFB ABC membrane transporters show brain location and transporter-specific maturation.

**Electronic supplementary material:**

The online version of this article (10.1007/s00418-020-01884-8) contains supplementary material, which is available to authorized users.

## Introduction

ATP-binding cassette (ABC) drug transporters form a vital part of the blood–brain barrier (BBB) and blood–cerebrospinal fluid barrier (BCSFB), where they mediate the efflux of a wide variety of chemical structures from endothelial and ependymal cells. Their specific localization and expression at the apical or basolateral plasma membrane impacts drug exposure in brain extracellular fluid (ECF) or cerebrospinal fluid (CSF) (Loscher and Potschka 2005; Morris et al. 2017).

ABC transporter localization has previously been established in adult human brain. P-glycoprotein (Pgp), breast cancer resistance protein (BCRP), and multidrug resistance-associated proteins (MRPs) 1, -2, -4, and -5 are expressed at the adult BBB (Morris et al. 2017; Nies et al. 2004). In addition, Pgp, MRP1 and MRP4 are present at the BCSFB, while this is debated for BCRP (Daood et al. 2008; Morris et al. 2017; Strazielle and Ghersi-Egea 2015). Together with multiple influx transporters, ABC transporters mediate brain disposition of various drugs and both influx and efflux have been described to affect brain ECF concentrations of transporter substrates (Morris et al. 2017; Strazielle and Ghersi-Egea 2015). The importance of ABC transporters at the BBB and BCSFB is supported by mouse knock-out models resulting in increased brain ECF exposure of morphine (Pgp knockout model), topotecan (BCRP and Pgp knockout model), and methotrexate (MRP4 knockout model) (Kanamitsu et al. 2017; Shen et al. 2009; Xie et al. 1999). Human BBB drug–drug interaction studies showed increased brain exposure of vinblastine after inhibition of Pgp by tariquidar (Bauer et al. 2015). Hence, it is likely that inter-individual variation in brain transporters expression or activity will result in altered brain drug exposure and resultant clinical effects (Billington et al. 2019).

In fetuses, neonates and children, BBB and BCSFB transporter expression levels are expected to be different as compared to adults, as this has previously been described for liver, kidney, and intestine (Cheung et al. 2019; Mooij et al. 2014; Prasad et al. 2016; van Groen et al. 2018). The limited fetal and neonatal studies available suggest low BBB Pgp expression in fetuses, which increases after birth, stable BCRP expression, and the absence of MRP1 expression (Daood et al. 2008; Lam et al. 2015). BCSFB staining was positive in children for Pgp and MRP1, but not for BCRP (Daood et al. 2008). As the existing data are mainly restricted to (young) fetuses, we now aim to study the developmental patterns for the ABC transporters Pgp, BCRP, and MRP 1, -2, -4, and -5 in a unique cohort ranging from fetus to infancy when compared to adults.

## Materials and methods

### Brain samples

The Erasmus MC Research Ethics Board waived the need for formal ethics approval according to the Dutch Law on Medical Research in Humans. Brain cortex and brain ventricle/choroid plexus tissue was collected when parental written informed consent for both autopsy and the explicit use of the tissue for research was present. The samples were selected when the tissue was morphologically normal. Tissues were derived from stillbirths or patients who passed away postnatally due to a wide variety of underlying conditions, which may have influenced brain function and possibly also BBB and/or BCSFB transporter protein expression. Examples of these conditions include, intrauterine infections, congenital anomalies and intrauterine growth retention, necrotizing enterocolitis, and heart disease. Human adult kidney tissue samples were a gift from Bart Smeets, PhD (Radboud University Medical Center, Nijmegen, The Netherlands) and human placental tissue samples from Rick Greupink, PhD (Radboud University Medical Center, Nijmegen, the Netherlands). These had been collected anonymously as (surgical) waste material. For these samples, a no-objection clause permitted use for research purposes in line with the Dutch guidelines on secondary use of human tissue.

### Immunohistochemistry

The formalin fixed, paraffin-embedded tissue blocks were cut in 4 μm slices and mounted on positively charged glass slides. The slides of post mortem tissues were heated for 25 min at 55 °C, deparaffinized and rehydrated in xylene and a series of ethanol. Depending on the transporter, antigen retrieval was performed with citrate buffer (pH6): Pgp, BCRP and MRP5 (“AMF”), or Tris–EDTA buffer (pH 9): MRP1, MRP2, MRP4, and MRP5 (“M5II-54”). Slides were incubated with a solution of 2% H2O2 (7047, Baker analyzed, Deventer, The Netherlands) in phosphate buffered saline (PBS)/Tween (0.1% Tween20 (T)) for half an hour, to quench endogenous peroxidase. Nonspecific binding was blocked using a solution of 1% bovine serum albumin (BSA) in PBS/T for 1 h and slides were incubated with primary antibody (dilutions and manufacturers shown in Table 1) in PBS/T 1% BSA at 4 °C overnight in a humidified container. Negative controls were incubated with a solution of PBS/T 1% BSA without primary antibody. Secondary antibodies (Table 1) were diluted 500× in PBS and slides were incubated for 30 min. Avidin–biotin–HRP complex (ABC-kit, Vectastain elite PK-6100, Vector Laboratories, Burlingame, United States) incubation was performed for 30 min to amplify the staining intensity. A dilution of 3,3′-diaminobenzidine (DAB D4168, Sigma-Aldrich, Darmstadt, Germany) was applied to the tissue, rinsed with tap water, and counter stained with hematoxylin. After a new series of alcohol to dehydrate tissue, and immersion in xylene, slides were mounted with xylene-based mounting medium Mountex (41-4021-00, MEDITE, Orlando, United States). IHC experiments were performed using a single batch of antibody and staining of all individual patient samples for a transporter protein of interest (e.g., Pgp) was performed in a single experiment in one run.

**Table 1:** Antibodies used

Protein	Primary antibody	Antigen retrieval	Dilution	Supplier
Pgp	HPA002199 (host: rabbit)	Citrate buffer (pH 6)	1:200	Sigma-Aldrich
BCRP	BXP-21 (host: mouse)	Citrate buffer (pH 6)	1:250	Kamiya biomedical
MRP1	QCRL-1 (host: mouse)	Tris–EDTA (pH 9)	1:66	Sigma-Aldrich
MRP2	M2 III-6 (host: mouse)	Tris**–**EDTA (pH 9)	1:50	Millipore
MRP4	M4I-80 (host: rat)	Tris–EDTA (pH 9)	1:20	Abcam
MRP4	M4I-10 (host: rat)	Tris–EDTA (pH 9)	1:75	Santa Cruz
MRP5	M5II-54 (host: rat)	Tris–EDTA (pH 9)	1:25	ThermoFisher
MRP5	AMF (host: rabbit)	Citrate buffer (pH 6)	1:20	Jedlitschky et al. (2000), Nies et al. (2004)

	Secondary antibody	Dilution	Supplier
Rabbit anti-mouse	AS09 609	1:500	Agrisera
Swine anti-rabbit	E 0431	1:500	DAKO
Rabbit anti-rat	R1371B	1:500	Origen

In case a transporter protein could not be detected in brain, kidney cortex (Pgp, BCRP, MRP1, -2, -4), and placenta tissue (MRP5) were used as positive controls, because these tissues show expression for all transporters investigated. The same procedure as described for brain samples was used, except that kidney material was blocked with an avidin and a biotin solution for 15 min, as kidney has high endogenous biotin expression.

### Microscopic imaging and quantification

Slides were scanned using the Slide Scanner Pannoramic 250 (3D Histech, brightfield, 3CCD camera, 45× magnification) and visualized using Pannoramic viewer version 1.15.2. Staining intensity was assessed separately for brain microvessels in the cortex regions (BBB), choroid plexus, and ventricular ependyma (both BCSFB). Subsequently, slides were scored: negative (0), detectable (1), intermediate (2) or high (3) by two independent observers (LV, AH) as previously performed by Daood et al. (2008), and scores were averaged in case of a discrepancy between scored values. Staining intensity scores were represented in scatterplots and a grouped analysis was done as performed by Cheung et al. using the age groups: preterm newborns (PNA 0–28 days; GA < 37 weeks), newborns (PNA 0–28 days), infants (1–24 months), children (2–12 years), and adults (> 16 years) (Cheung et al. 2019). Owing to the low amount of samples, the age groups infants and children were combined.

### Statistics

The data were described using standard statistics. To test for correlations between staining intensity score and postmenstrual-, postnatal-, and gestational age (PMA, PNA, and GA), a Spearman’s rank correlation coefficient was calculated using Graphpad Prism version 5.03.

## Results

### Patient characteristics

Post mortem brain cortex tissue derived from 23 fetuses (1 first trimester, 10 second trimester, and 12 third trimester, GA range 12.9–39 weeks), 17 neonates (GA range 24.6–41.3 weeks, PNA range 0.004–3.5 weeks), 8 children (PNA range 0.1–3 years), and 4 adults were obtained from the Erasmus MC Tissue Bank, Rotterdam. For 15 fetuses and children ventricle/choroid plexus tissue (BCSFB) was available.

### Transporter localization and expression

#### P-glycoprotein

**Fig. 1:**
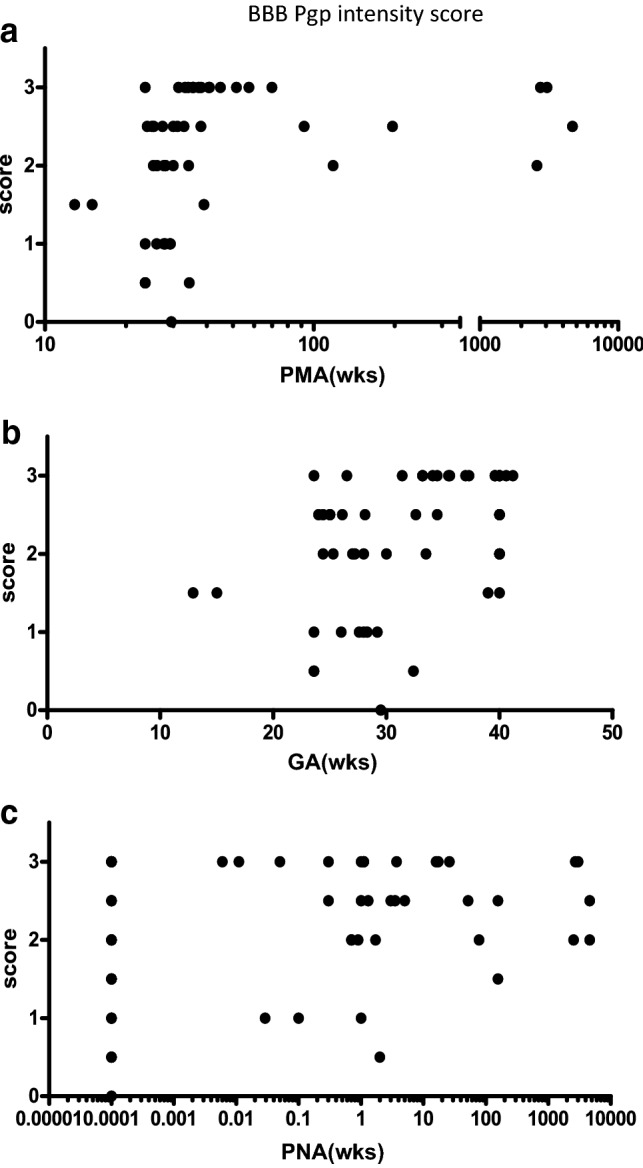
Pgp staining intensity scores in BBB against **a** postmenstrual age, **b** gestational age, and **c** postnatal age. wks indicates age in weeks

BBB: An age dependent increase was observed for Pgp staining intensity score as a function of PMA, GA and PNA in cortex microvessels (Fig. 1; Table 2). Samples showing low or no staining were mainly from individuals with a PMA between 13 and 40 weeks.

**Table 2:** Correlation of BBB staining intensity score with age

Transporter protein	PMA	GA	PNA
Pgp	*ρ* = **0.5351**, *p* = < 0.0001	*ρ* = **0.4423**, *p* = 0.0009	*ρ* = **0.2854**, *p* = 0.0383
BCRP	*ρ* = **0.4303**, *p* = 0.0013	*ρ* = **0.4350**, *p* = 0.0011	*ρ* = 0.2604, *p* = 0.0597
MRP1	*ρ* = − 0.1164, *p* = 0.4209	*ρ* = − 0.1052, *p* = 0.4670	*ρ* = − 0.1988, *p* = 0.1663
MRP2	*ρ* = − 0.1556, *p* = 0.2708	*ρ* = − 0.06476, *p* = 0.6450	*ρ* = − 0.2340, *p* = 0.0917

*PMA* postmenstrual age, *GA* gestational age, *PNA* postnatal age

*ρ* = Spearman’s correlation coefficient. Bold = statistically significant (*p* < 0.05)

**Fig. 2:**
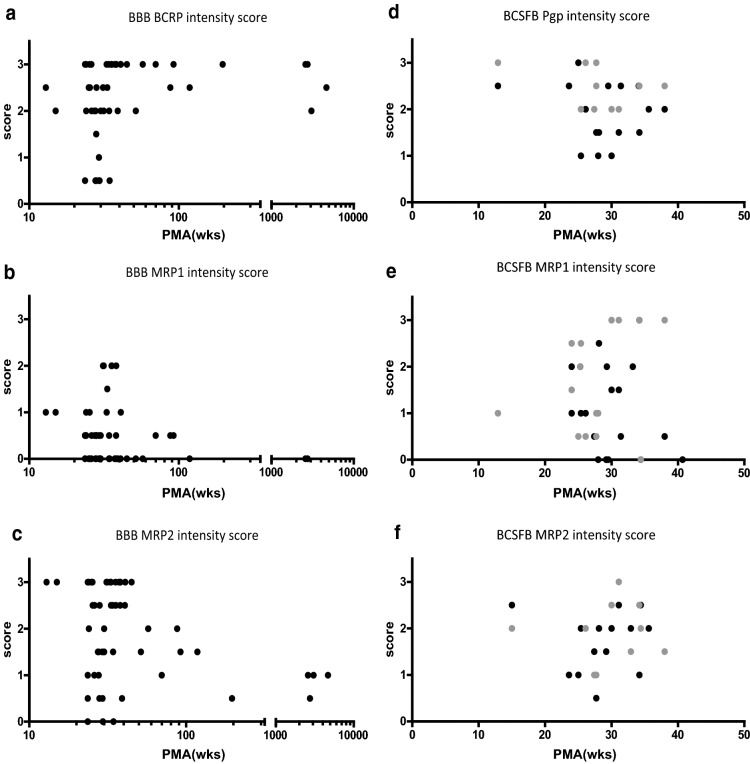
Postmenstrual age (PMA) versus staining intensity score in BBB for **a** BCRP, **b** MRP1, **c** MRP2 and postmenstrual age versus staining intensity score in BCSFB for **d** Pgp, **e** MRP1, **f** MRP2. In panel **d** black dots indicate scoring of ventricle ependymal cells and gray dots scoring of choroid plexus. wks indicates age in weeks

**Fig. 3:**
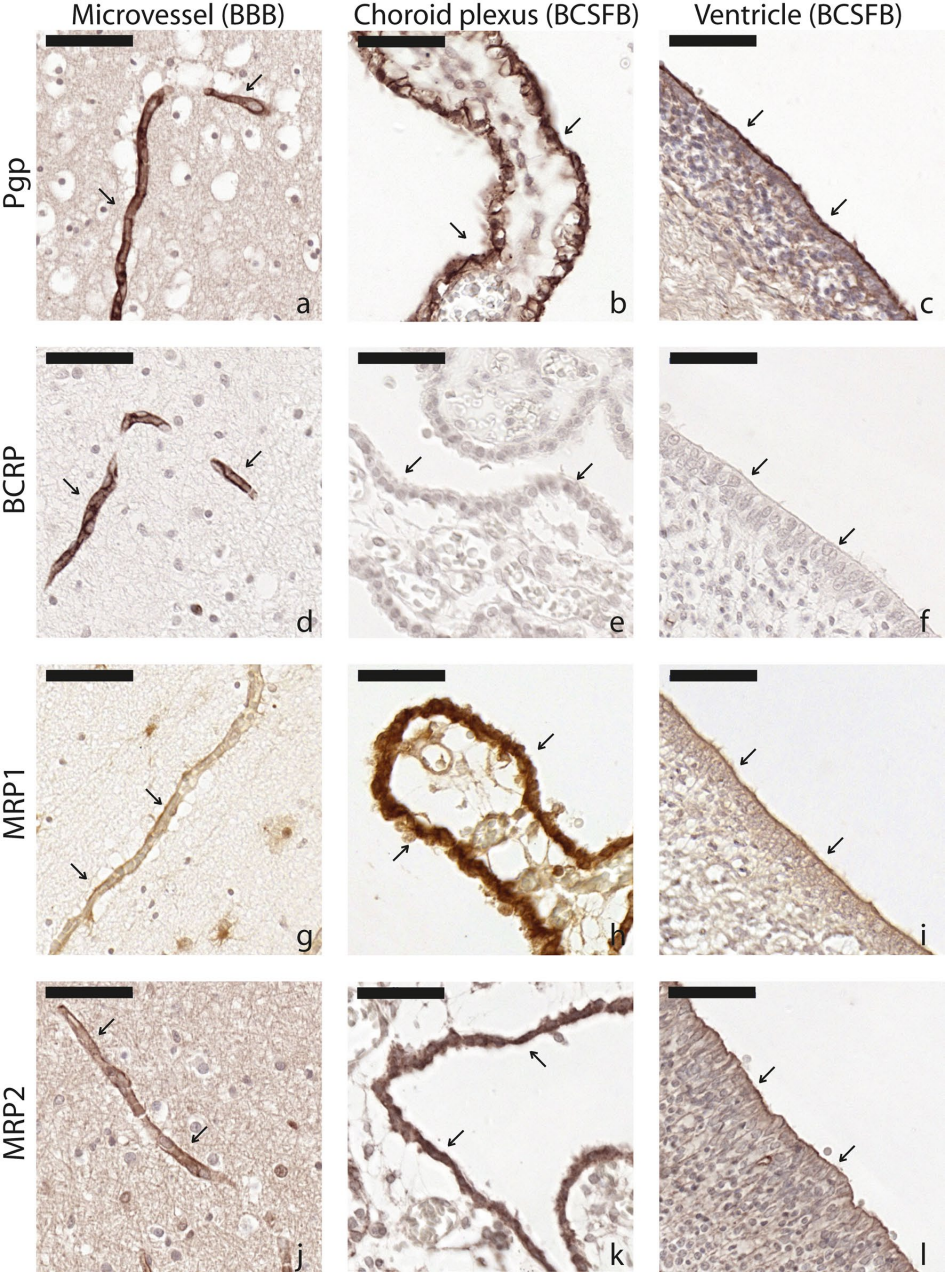
Representative images from fetal and neonatal brain samples for Pgp, BCRP, MRP1, and MRP2 in microvessels (**a**, **d**, **g**, and **j**), choroid plexus (**b**, **e**, **h**, and **k**), and ventricle (**c**, **f**, **i**, and **l**). All tissues were from children < 45 weeks PMA. Arrows highlight locations of interest. Scale bar 50 μm

BCSFB: Positive staining was observed for choroid plexus and ventricular ependymal cells as early as 12.9 weeks PMA. No age-related differences in staining intensity were observed (Fig. 2). Staining in ventricle ependymal cells was restricted to the apical membrane (Fig. 3).

#### Breast cancer resistance protein

BBB: BCRP-staining intensity related to age as a function of PMA and GA (Fig. 2, Table 2). Staining variability was especially marked around 13–40 weeks PMA. In this age period, staining intensity was low in several samples, which was not seen in older children and adults.

BCSFB: No staining was detected in choroid plexus or ventricle ependymal cells (Fig. 3).

#### Multidrug resistance-associated protein 1

BBB: MRP1 staining was weakly positive in part of the brain cortical vessels in fetal and pediatric samples (between 13 and 40 weeks PMA), but not in adults. For a few samples, clear staining of vessels could be seen at a PMA between 30 and 40 weeks (Figs. 2, 3).

BCSFB: Staining was pronounced in choroid plexus and ventricular epithelium, but no age-related effects could be observed at this barrier. In ventricular epithelium, staining could be observed only at the apical membrane (Figs. 2, 3).

#### Multidrug resistance-associated protein 2

BBB: For most samples, staining intensity of MRP2 appeared higher until 100 weeks PMA, as compared to adult material by visual inspection (Fig. 2). No significant correlation was observed between MRP2 and age (Table 2).

BCSFB: MRP2 staining was positive in choroid plexus and ventricle wall ependymal cells, but no developmental pattern could be seen. The expression in ventricle epithelium was detected at the apical side of the ependymal cells (Figs. 2, 3).

#### Multidrug resistance-associated protein 4

MRP4 could not be detected in brain cortex microvessels, choroid plexus, or ventricular ependymal cells (data not shown). MRP4 was detected in positive control kidney cortex material using two different antibodies (Online Resource 1).

#### Multidrug resistance-associated protein 5

Staining in brain cortex microvessels was only positive in erythrocytes in these vessels. Staining in microvessel endothelial cells, choroid plexus, or ventricle was not detected (data not shown). MRP5 was stained in placental tissue used as positive control with two different antibodies (Online Resource 1).

### Gestational and postnatal age versus transporter expression

Online Resources 2 and 3 present the staining intensity of the brain cortex BBB transporters BCRP, MRP1 and MRP2 and BCSFB transporters Pgp, MRP1, and MRP2 in relation to postnatal and gestational age.

### Grouped analysis of brain cortex BBB transporter expression

Online Resource 4 presents the staining intensity of the brain cortex BBB transporters Pgp, BCRP, MRP1, and MRP2 in the age groups defined previously.

## Discussion

Our results show both location as well as transporter-specific variation in ABC transporter expression in young children as compared to adults. Pgp, BCRP, MRP1, and MRP2 were detected in the cortical BBB, although not across the complete age range. Staining intensity was lower for Pgp and BCRP in brain cortex microvessels from fetuses and neonates, as compared to adults. In contrast, MRP1 and MRP2 staining intensity appeared higher in fetuses, neonates and children, but a correlation with age was not statistically significant. Choroid plexus and ventricle wall ependymal cells were positively stained for Pgp, MRP1, and MRP2 and intensity appeared stable. Transporter expression detected in ventricle was present at the apical side of ependymal cells for Pgp, MRP1 and MRP2, whereas plasma membrane localization could not be specified in microvessels and choroid plexus. MRP4 and MRP5 could not be detected in brain cortex microvessels, ventricular epithelium, and choroid plexus.

### P-glycoprotein

Pgp expression has previously been described in brain cortex microvessels forming the BBB in fetal, pediatric, and adult animals and humans (Daood et al. 2008; Lam et al. 2015; Tsai et al. 2002; Uchida et al. 2011). In addition, weak Pgp staining of pericytes and astrocytes has been described in some studies, but this was not observed in our samples possibly due to the relatively high dilution of primary antibody (Virgintino et al. 2002). Our observed increase in the expression with increasing age is consistent with the literature and appeared mature at 40 weeks postmenstrual age and less than 7 days PNA. In contrast, Lam et al. suggested full maturation around 3–6 months PNA (Lam et al. 2015). Our study is consistent with the results of Daood et al. where Pgp expression increased between 22 and 42 weeks gestational age. Lower Pgp BBB expression in fetuses and neonates might result in an increase in brain ECF concentrations of Pgp substrates. For example, this mechanism could explain the increased risk of respiratory depression with morphine in neonates (Taddio et al. 2009). BCSFB expression of Pgp is present at the apical cell membrane in adults (Morris et al. 2017). This was also observed in our study for cells in the ventricle ependyma, which belong to the BCSFB. Reduced apical Pgp activity will result in a reduced efflux of substrates into the CSF. This is supported by decreased CSF concentrations in a mouse Pgp knockout model with topotecan as Pgp (and BCRP) substrate (Shen et al. 2009).

### Breast cancer resistance protein

BCRP cortical BBB staining intensity was variable, ranging from low to adult levels in children between 13 weeks and 40 weeks PMA, after which expression became more consistently intense. This is in contrast with a previous study in which fetal and adult staining was similar (Daood et al. 2008). Our study provides the first indication that dosing of BCRP substrates might result in higher brain ECF exposures due to lower BCRP expression in the fetal and neonatal age range. The lack of BCRP staining in choroid plexus and ventricle wall ependymal cells was similar across the complete age range and consistent with low or no expression in human and animal studies (Daood et al. 2008; Eisenblatter et al. 2003; Reichel et al. 2011).

### Multidrug resistance-associated protein 1

In our study, pediatric MRP1 expression was detected in brain cortex BBB of some fetuses and children, in contrast to the study by Daood et al. (2008). Interestingly, others also found low or no MRP1 expression levels in adults using immunohistochemistry or western blotting (Aronica et al. 2004; Nies et al. 2004; Regina et al. 1998). Consistent with the previous findings was the intense staining of MRP1 in the choroid plexus and ventricle wall ependymal cells (Daood et al. 2008).

### Multidrug resistance-associated protein 2

MRP2 cortical BBB expression had an inverse pattern as compared to the developmental pattern seen for Pgp. This is consistent with studies in adult brain tissue, where generally no expression was seen and only in case of epilepsy or associated co-medication MRP2 becomes upregulated (Aronica et al. 2004; Dombrowski et al. 2001; Luna-Munguia et al. 2015). This is the first time that BBB expression has been evaluated in a pediatric population and that intense staining intensity was observed for pediatric samples. MRP2 was also detected in choroid plexus ependymal cells and ventricle ependymal cells.

### Multidrug resistance-associated protein 4 and 5

MRP4 and MRP5 were not detected in cortical BBB epithelial cells or BCSFB ependymal cells. MRP5 was stained in erythrocytes retained in the microvessels, which confirms earlier reports of MRP5 expression in red blood cells (Jedlitschky et al. 2000). This is in contradiction to findings by Nies et al. who described MRP4 and MRP5 staining in microvessels, and also with proteomic analysis of brain microvessels and the brain endothelial hCMEC/D3 cell line, in which MRP4 was detected (Nies et al. 2004; Ohtsuki et al. 2013; Uchida et al. 2011). These differences can potentially be explained by the disease state or previous drug treatment in the study by Nies et al., as this is known to influence transporter expression levels (Gu and Manautou 2010; Nies et al. 2004). Also, cell culture conditions affect transporter expression levels. In our study, post mortem material was obtained from a heterogenous patient population.

If we compared BBB and BCSFB expression with transporter expression in other organs, it becomes apparent that ontogeny is transporter and organ dependent. Pgp consistently shows low to high expression while age increases in brain, kidney and liver, but remains stable in intestine (Cheung et al. 2019; Daood et al. 2008; Konieczna et al. 2011; Prasad et al. 2016; van Groen et al. 2018). For MRP1 and MRP2 a trend towards a high to low expression profile as a function of PMA was observed in this study, whereas an age-related increase in liver expression is described (van Groen et al. 2018). Ontogeny in MRP2 kidney expression appears stable, while MRP1 remained undetected (Cheung et al. 2019). In addition, BCRP expression was relatively stable in kidney and liver (Cheung et al. 2019; van Groen et al. 2018). An important aspect to consider is whether to correlate transporter expression with postnatal age, gestational age or postmenstrual age. This will be dependent on factors driving expression, such as changes in transcription factors, epigenetics and hormones, and the moment they become important during development (e.g., as a consequence of birth). To date little is known about factors mediating transporter expression differences (Cheung et al. 2019).

A limitation of our study is the use of immunohistochemical staining to assess an age-related variation in ABC efflux transporter expression. As this method only allows semi-quantitative analysis, we were able to describe developmental patterns, but not quantitative differences. Moreover, as discussed before, disease conditions, medication, delay between death and autopsy, and IHC methodology (e.g., methods of fixation) may have influenced the identified age-related variation (Brouwer et al. 2015; Nicolas and de Lange 2019). Reports have indicated that especially inflammation and ischemia influence transporter expression in transporter protein and organ dependent fashion (Evers et al. 2018). In addition, some drugs act intracellularly and therefore the plasma membrane barrier needs to be passed at which transporters might influence their disposition (Bendayan et al. 2006; Lee et al. 2001; Scism et al. 2000; Wang and Welty 1996). The limitations in these human studies warrant careful interpretation of the findings. To support our findings, the expression patterns for Pgp increased with age, consistent with the previous literature. This suggests that despite its limitations, the material we studied appeared suitable to investigate age-dependent trends in the expression patterns of Pgp and likely other ABC transporter proteins.

In conclusion, this study indicates that Pgp and BCRP BBB expression is low and more variable around birth, potentially resulting in higher brain ECF concentrations of transporter substrates, whereas MRP1 and MRP2 expression appeared high around birth, decreasing towards adulthood.

## Electronic supplementary material

Below is the link to the electronic supplementary material.

**ESM_1** MRP4 expression in kidney cortex tissue **a** (M4I-80), **b** (M4I-10), and MRP5 expression in placental tissue **c** (M5II-54), **d** (AMF). Arrows indicate localization of the protein of interest. Magnification is 400x (TIFF 38049 kb)

**ESM_2** Postnatal age versus staining intensity score in BBB for **a**: BCRP, **b**: MRP1, **c**: MRP2, and postnatal age versus staining intensity score in BCSFB for **d**: Pgp, **e**: MRP1, and **f**: MRP2. In panel d black dots indicate scoring of ventricle ependymal cells and gray dots scoring of choroid plexus. wks indicates age in weeks (EPS 1337 kb)

**ESM_3** Gestational age versus staining intensity score in BBB for **a**: BCRP, **b**: MRP1, **c**: MRP2, and gestational age versus staining intensity score in BCSFB for **d**: Pgp, **e**: MRP1, and **f**: MRP2. In panel d black dots indicate scoring of ventricle ependymal cells and gray dots scoring of choroid plexus. wks indicates age in weeks (EPS 1305 kb)

**ESM_4** Staining intensity (mean and SEM) of the brain cortex BBB transporters **a**: Pgp, **b:** BCRP, **c:** MRP1, and **d**: MRP2 in the age groups: preterm newborns (PNA 0–28 days; GA < 37 weeks), newborns (PNA 0–28 days), infants/children (1 month–12 years), and adults (> 16 years) (EPS 1070 kb)
