# Supplementary figures and images for: Developmental patterns in human blood–brain barrier and blood–cerebrospinal fluid barrier ABC drug transporter expression
Source: Histochem Cell Biol. 2020 May 24;154(3):265–73. doi: 10.1007/s00418-020-01884-8 (PMC7502061; doi:10.1007/s00418-020-01884-8)

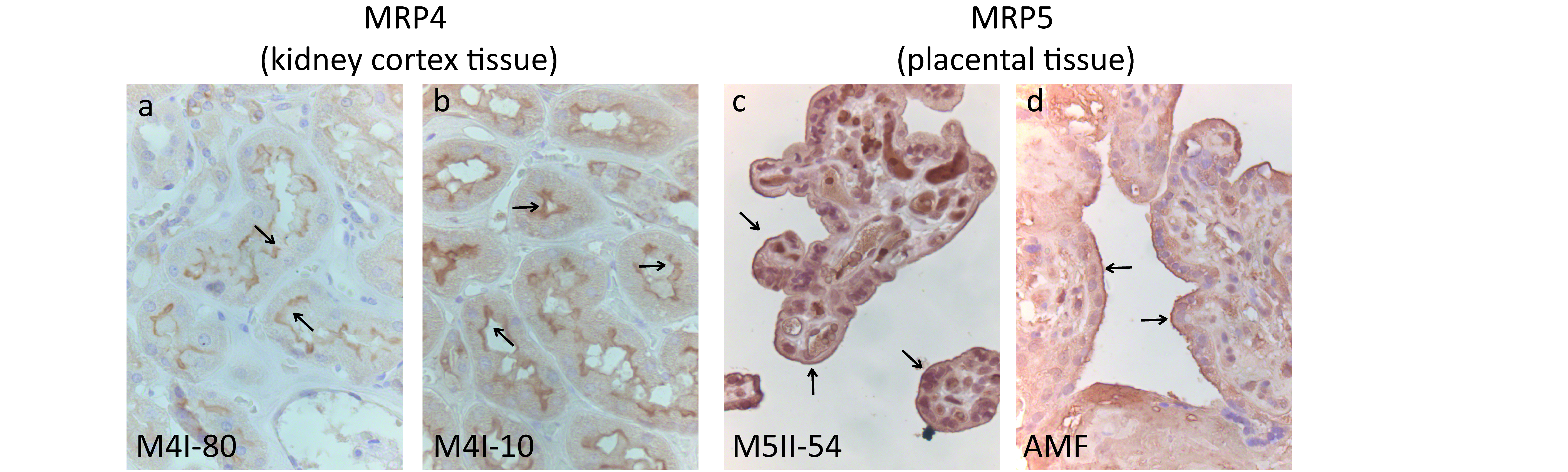

Supplement: Supplementary file 1 — ESM_1 MRP4 expression in kidney cortex tissue a (M4I-80), b (M4I-10), and MRP5 expression in placental tissue c (M5II-54), d (AMF). Arrows indicate localization of the protein of interest. Magnification is 400x (TIFF 38049 kb) [file 418_2020_1884_MOESM1_ESM.tif]
